# Functional redundancy of ubiquitin-like sulfur-carrier proteins facilitates flexible, efficient sulfur utilization in the primordial archaeon *Thermococcus kodakarensis*

**DOI:** 10.1128/mbio.00534-24

**Published:** 2024-07-08

**Authors:** Ryota Hidese, Takayuki Ohira, Satsuki Sakakibara, Tsutomu Suzuki, Naoki Shigi, Shinsuke Fujiwara

**Affiliations:** 1 Graduate School of Science, Technology and Innovation, Kobe University, Kobe, Japan; 2 Department of Chemistry and Biotechnology, Graduate School of Engineering, The University of Tokyo, Tokyo, Japan; 3 Department of Bioscience, Graduate School of Science and Technology, Kwansei-Gakuin University, Sanda, Hyogo, Japan; 4 Computational Bio Big-Data Open Innovation Laboratory, National Institute of Advanced Industrial Science and Technology (AIST), Tokyo, Japan; New York University School of Medicine, New York, New York, USA; University of Illinois at Urbana Champaign, Urbana, Illinois, USA

**Keywords:** ubiquitin-like protein, thermophilic archaea, molybdopterin, tungstopterin, tRNA thionucleoside, elemental sulfur

## Abstract

Ubiquitin-like proteins (Ubls) in eukaryotes and bacteria mediate sulfur transfer for the biosynthesis of sulfur-containing biomolecules and form conjugates with specific protein targets to regulate their functions. Here, we investigated the functions and physiological importance of Ubls in a hyperthermophilic archaeon by constructing a series of deletion mutants. We found that the Ubls (TK1065, TK1093, and TK2118) in *Thermococcus kodakarensis* are conjugated to their specific target proteins, and all three are involved in varying degrees in the biosynthesis of sulfur-containing biomolecules such as tungsten cofactor (Wco) and tRNA thiouridines. TK2118 (named UblB) is involved in the biosynthesis of Wco in a glyceraldehyde 3-phosphate:ferredoxin oxidoreductase, which is required for glycolytic growth, whereas TK1093 (named UblA) plays a key role in the efficient thiolation of tRNAs, which contributes to cellular thermotolerance. Intriguingly, in the presence of elemental sulfur (S^0^) in the culture medium, defective synthesis of these sulfur-containing molecules in Ubl mutants was restored, indicating that *T. kodakarensis* can use S^0^ as an alternative sulfur source without Ubls. Our analysis indicates that the Ubl-mediated sulfur-transfer system in *T. kodakarensis* is important for efficient sulfur assimilation, especially under low S^0^ conditions, which may allow this organism to survive in a low sulfur environment.

**IMPORTANCE:**

Sulfur is a crucial element in living organisms, occurring in various sulfur-containing biomolecules including iron-sulfur clusters, vitamins, and RNA thionucleosides, as well as the amino acids cysteine and methionine. In archaea, the biosynthesis routes and sulfur donors of sulfur-containing biomolecules are largely unknown. Here, we explored the functions of Ubls in the deep-blanched hyperthermophilic archaeon, *Thermococcus kodakarensis*. We demonstrated functional redundancy of these proteins in the biosynthesis of tungsten cofactor and tRNA thiouridines and the significance of these sulfur-carrier functions, especially in low sulfur environments. We propose that acquisition of a Ubl sulfur-transfer system, in addition to an ancient inorganic sulfur assimilation pathway, enabled the primordial archaeon to advance into lower-sulfur environments and expand their habitable zone.

## INTRODUCTION

**Fig 1:**
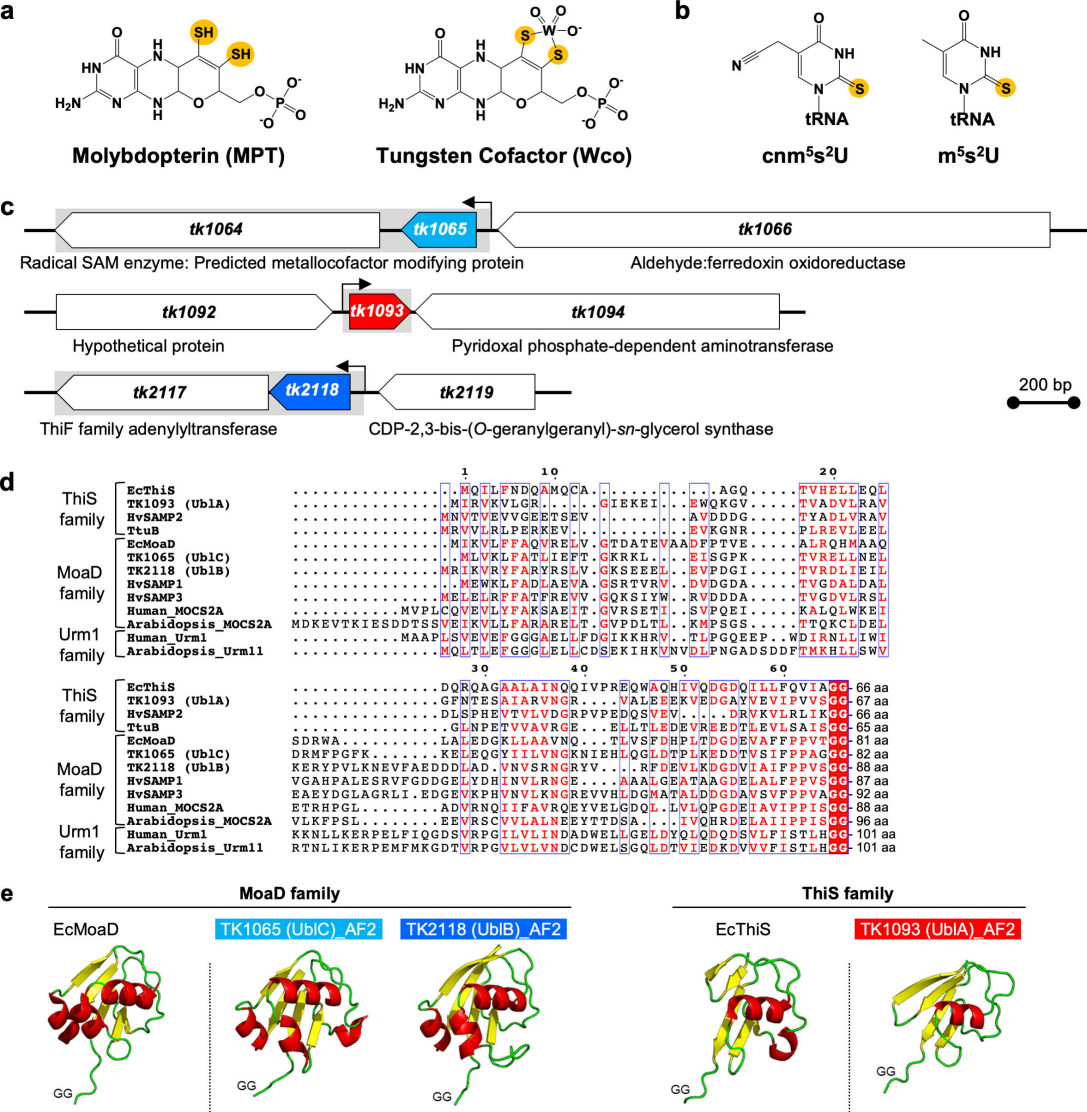
Ubls in *T. kodakarensis*. (**a**) Chemical structures of MPT (left) and Wco (right). (**b**) Chemical structures of thionucleosides in tRNA. (**c**) Gene structure of *ubl* genes *tk1065*, *tk1093*, and *tk2118* in the *T. kodakarensis* genome. The gray box and black arrow indicate a transcription unit with a promoter, respectively. (**d**) Multiple amino acid sequence alignment analysis of Ubl orthologs generated using the MUSCLE algorithm with MEGAX and visualized by Espript version 3.0 (https://espript.ibcp.fr/ESPript/ESPript/). White and red letters in blue boxes indicate identical and similar residues, respectively. The length of each protein is shown on the right side of the alignment. UniProt accession numbers for the sequences are TK1065 (UblC), Q5JE16; TK1093 (UblA), Q5JE22; TK2118 (UblB), Q5JEX1; *Escherichia coli* MoaD (EcMoaD), P30748; *E. coli* ThiS (EcThiS), O32583; SAMP1 from *Haloferax volcanii* (HvSAMP1), D4GUF6; SAMP2 from *H. volcanii* (HvSAMP2), D4GZE7; SAMP3 from *H. volcanii* (HvSAMP3), D4GVB0; TtuB from *Thermus thermophilus* HB27, Q72LF4; MOCS2A from *Homo sapiens* (Human_MOCS2A), O96033; Urm1 from *H. sapiens* (Human_Urm1), Q9BTM9; MOCS2A from *Arabidopsis thaliana* (Arabidopsis_MOCS2A), Q9S7A3; Urm11 from *A. thaliana* (Arabidopsis_Urm11), A0MDQ1. (**e**) Ribbon diagrams of the three-dimensional structures of EcThiS (PDB entry 1ZUD) and EcMoaD (PDB entry 1JW9), and structural models of TK1093 (UblA), TK1065 (UblC), and TK2118 (UblB) from the AlphaFold database, hosted at European Molecular Biology Laboratory–European Bioinformatics Institute (https://alphafold.ebi.ac.uk). Figures were prepared using PyMOL software (http://pymol.org/pymol). Confidence values of Alphafold models are shown in Fig. S1c.

Bacteria, eukaryotes, and some archaea utilize L-cysteine as a sulfur source for the biosynthesis of sulfur-containing biomolecules, including iron-sulfur clusters, molybdopterin (MPT, Fig. 1a), and tRNA thionucleosides (Fig. 1b), which are vital for all domains of life (1–5). The sulfur atom of L-cysteine is transferred to the final recipient biomolecule through sulfur-transfer pathways composed of many sulfur-carrier proteins (6, 7). In the first step, cysteine desulfurase (CD) binds the sulfur atom of L-cysteine to form an enzyme-bound persulfide, without releasing harmful reactive sulfur species in solution. The terminal sulfur of the persulfide in CD is subsequently transferred to sulfur-carrier proteins. There exist two types of sulfur carrier: (1) protein persulfide (R-SSH) exemplified by CD and rhodanese homology domain-containing proteins with a conserved cysteine residue; and (2) protein thiocarboxylate (R-COSH) formed at conserved C-terminal di-glycine residues of Ubls (8). Finally, the sulfur atoms of sulfur-carrier proteins are incorporated into target molecules by specific sulfurtransferases in each biosynthesis pathway.

Although these sulfur-transfer systems are widely conserved in all domains of life, many hyperthermophilic archaea do not possess CD orthologs (Fig. S1a) (9). Many live under inorganic sulfur compound-rich conditions and are assumed to take environmental inorganic sulfur into their cytoplasm and utilize it as a sulfur source directly for final sulfurtransferases in biosynthesis pathways of sulfur-containing biomolecules (9). However, the facultatively sulfur-reducing hyperthermophilic archaeon *Thermococcus kodakarensis* does possess a CD ortholog (Fig. S1a). CD is dispensable for iron-sulfur cluster biosynthesis and cell growth in the presence of elemental sulfur (S^0^, cyclo-octasulfur) (5), suggesting that *T. kodakarensis* can use both L-cysteine and environmental S^0^ as sulfur sources. However, unlike CDs, sulfur-carrier protein Ubls are highly conserved in archaea (10), although their functions remain largely unknown.

Here, we characterized the molecular functions and biological importance of three Ubls (TK1065, TK1093, and TK2118) of *T. kodakarensis* and found that these proteins act both as sulfur-carrier proteins and protein modifiers. TK2118 (named UblB) is required for growth with maltodextrin (Mdx) as a sole carbon source, suggesting that it mediates incorporation of tungsten cofactor (Wco) into the key enzyme, glyceraldehyde-3-phosphate:ferredoxin oxidoreductase (GOR), essential for glycolysis in the Mdx medium. TK1093 (UblA) mediates sulfur-transfer for the synthesis of tRNA thionucleosides and contributes to cell growth at high temperature. Moreover, we revealed that Ubls were partially dispensable for the synthesis of these sulfur biomolecules under S^0^-rich conditions. These results clearly indicate the presence of an inorganic sulfur assimilation pathway in *T. kodakarensis*, which may reflect the metabolic heritage of protobionts. The findings also suggest that acquisition of CDs and Ubls enables efficient synthesis of sulfur-containing biomolecules under low concentrations of sulfur donors, thereby allowing these organisms to advance into lower-sulfur environments and expand their habitable zone.

## RESULTS

### *T. kodakarensis* possesses three Ubl orthologs

Ubls are small proteins with a β-grasp fold and a conserved carboxy-terminal di-glycine motif (8, 11) that are classified into two distinct families in bacteria: ThiS and MoaD (4) (Fig. S1b). ThiS paralogs function as sulfur carriers for the biosynthesis of thiazole moiety of thiamin in eubacteria or thiouridine in tRNAs (4, 12) (Fig. 1b). Meanwhile, MoaD paralogs are sulfur carriers for the biosynthesis of MPT moieties of molybdenum/tungsten cofactors Moco/Wco (4) (Fig. 1a). Homology searches showed that *T. kodakarensis* has three Ubl orthologs: *tk1065*, *tk1093*, and *tk2118* (11) (Fig. 1c through e; Fig. S1). As shown by phylogenic analysis (Fig. S1b) and structural comparison with AlphaFold 2 models (13) (Fig. 1e), TK1093 belongs to the ThiS family, whereas TK1065 and TK2118 belong to the MoaD family. The *tk1065* gene is located between genes encoding tungstopterin-containing aldehyde:ferredoxin oxidoreductase (AOR, *tk1066*) (14) and a radical *S*-adenosylmethionine (SAM) enzyme (*tk1064*), which might be involved in the maturation of the tungsten-containing cofactors in AOR (*tk1066*) (15, 16) (Fig. 1c). The *tk1065* gene likely forms an operon with *tk1064*, based on previous RNA sequencing analysis (17), whereas *tk1093* appears to be a monocistronic gene (17) (Fig. 1c). The *tk2118* gene is tandemly arranged and forms an operon with a homologous gene encoding ATP-dependent E1/MoeB/ThiF-type enzyme (*tk2117*, Fig. 1c), which adenylates the C-terminal glycine residue of Ubl to form the active intermediate (Ubl-COAMP) for the subsequent sulfur-transfer step (18). Indeed, we observed that TK2117 formed pyrophosphate in the presence of TK2118 (Fig. S2), which suggests that TK2117 catalyzes the adenylation of the C-terminus of TK2118. We named TK1065, TK1093, and TK2118 as UblC, UblA, and UblB, respectively, where “Ubl” stands for “ubiquitin-like protein.”

### Three Ubls contribute differently to the growth of *T. kodakarensis*, depending on temperature and sulfur availability

**Fig 2:**
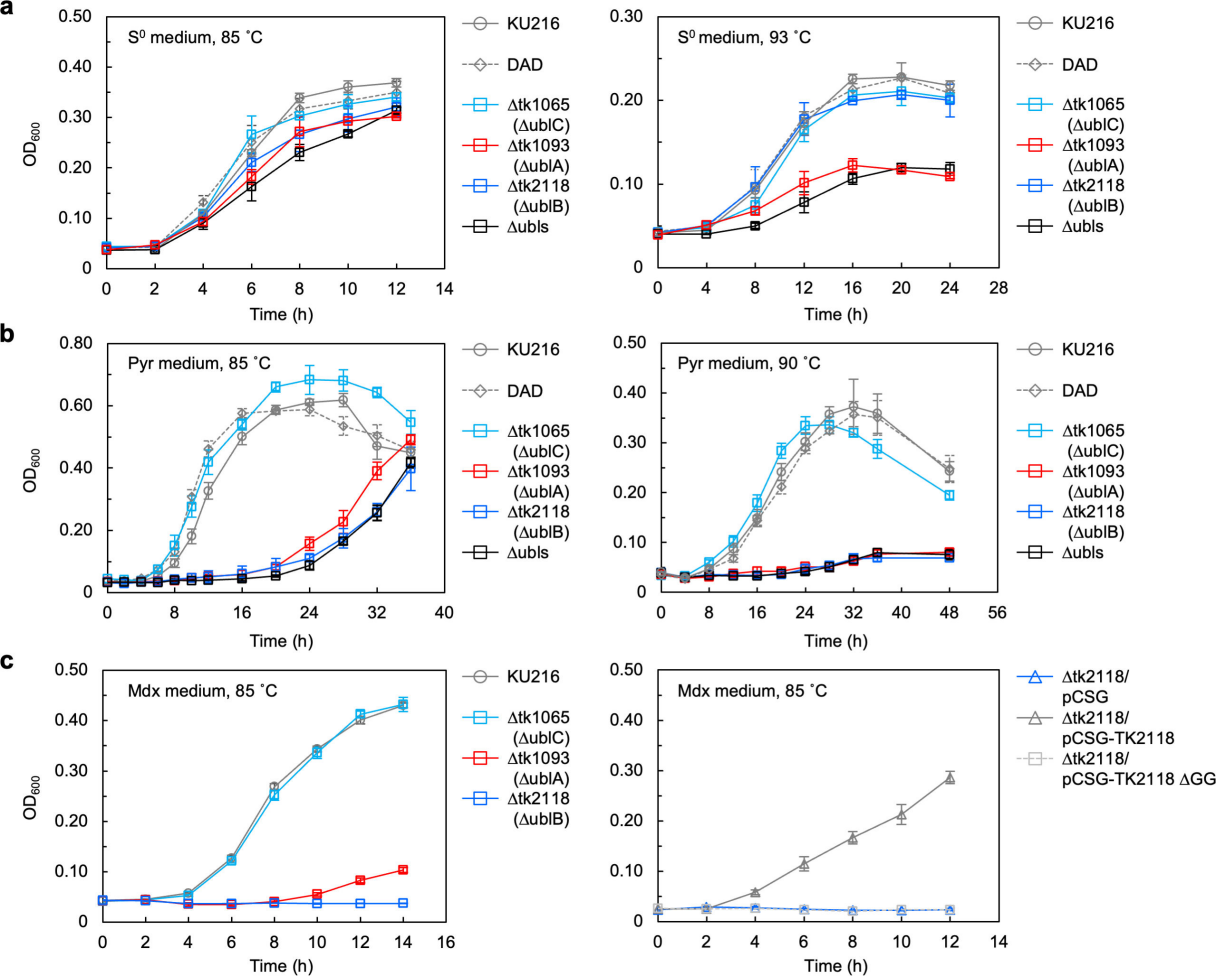
Growth characteristics of Ubl-deficient strains. (**a**) Growth comparison of *T. kodakarensis* strains in ASW-YT-S^0^ (S^0^ medium) at 85°C (left panel) or 93°C (right panel). Symbols: gray circles, *T. kodakarensis* KU216 strain; gray diamonds connected with broken lines, *T. kodakarensis* DAD strain; cyan squares, ∆ublC strain; red squares, ∆ublA strain; blue squares, ∆ublB strain; and black squares, ∆ubls strain. (**b**) ASW-YT-based medium supplemented with pyruvate (Pyr medium) at 85°C (left panel) or 90°C (right panel). Symbols are as in panel a. (**c**) (Left panel) The host strain and each Ubl-deficient strain were cultivated in ASW-YT medium supplemented with 0.2 wt/vol % maltodextrin (Mdx medium) at 85°C. Symbols are as in panel a. (Right panel) ∆tk2118 (∆ublB) with pCSG (blue triangles), pCSG-TK2118 (gray triangles), or pCSG-TK2118 ∆GG (light gray squares connected with broken lines) was cultivated with Mdx medium at 85°C. Error bars indicate the standard deviations of three independent culture experiments.

To uncover the biological functions of the three Ubl orthologs in *T. kodakarensis*, we constructed deletion mutants for each Ubl gene and a triple Ubl deletion mutant (∆ubls) from the parent strain DAD (∆*pyrF* and ∆*pdaD*; Fig. S3). The growth phenotypes of these strains on artificial seawater (ASW)-YT-S^0^ medium (S^0^-containing medium) at optimal (85°C) and high (93°C or 90°C) temperatures were examined (Fig. 2a). *T. kodakarensis* grows at temperatures ranging from 60°C to 95°C, with an optimum growth temperature of 85°C. Since wild-type strains grow slowly at 93°C on Pyr medium, the “high” temperature was set to 90°C when Pyr medium was used. At the optimal temperature, these strains showed almost the same growth rates. However, the ∆ublA strain showed growth retardation at 93°C compared with the parent strain and other Ubl-deficient strains. The ∆ubls strain also showed growth phenotypes similar to ∆ublA. In ASW-YT-Pyr medium (Pyr medium, no S^0^; Fig. 2b), ∆ublA and ∆ublB strains displayed severe growth retardation at optimal (85°C) and high (90°C) temperatures. The ∆ubls strain also exhibited similar growth defects to ∆ublA and ∆ublB strains. These results suggest that UblA and UblB are required for cell growth especially at higher temperature in *T. kodakarensis* in the absence of environmental S^0^, but their functions could be partially compensated for in the presence of environmental S^0^.

### The roles of Ubls in MPT biosynthesis

MPT consists of a pyranopterin, a complex heterocycle featuring a pyran fused to a pterin ring, and the pyran ring contains two thiolates that serve as ligands for a molybdenum or a tungsten atom to form Moco or Wco, respectively (Fig. 1a). Moco and Wco are organometallic cofactors, which are essential for the activities of various oxidoreductases (19–24). GOR, a glyceraldehyde 3-phosphate:ferredoxin oxidoreductase mediating an essential step in maltose fermentation, requires Wco as a cofactor (24–26). To investigate the requirement of each Ubl for the synthesis of GOR-incorporated Wco, the strains were cultured in ASW-YT-based medium supplemented with maltodextrin (Mdx medium, no S^0^). In Mdx medium, the ∆ublB strain was unable to grow, while the ∆ublA strain showed severe growth retardation (Fig. 2c), as observed in Pyr medium (Fig. 2b). The growth defect of the ∆ublB strain was restored by ectopic expression of the wild-type *ublB* gene but not the *ublB* gene lacking the C-terminal two glycine residues (Fig. 2c), suggesting that UblB and its C-terminus are essential for MPT synthesis in GOR.

**Fig 3:**
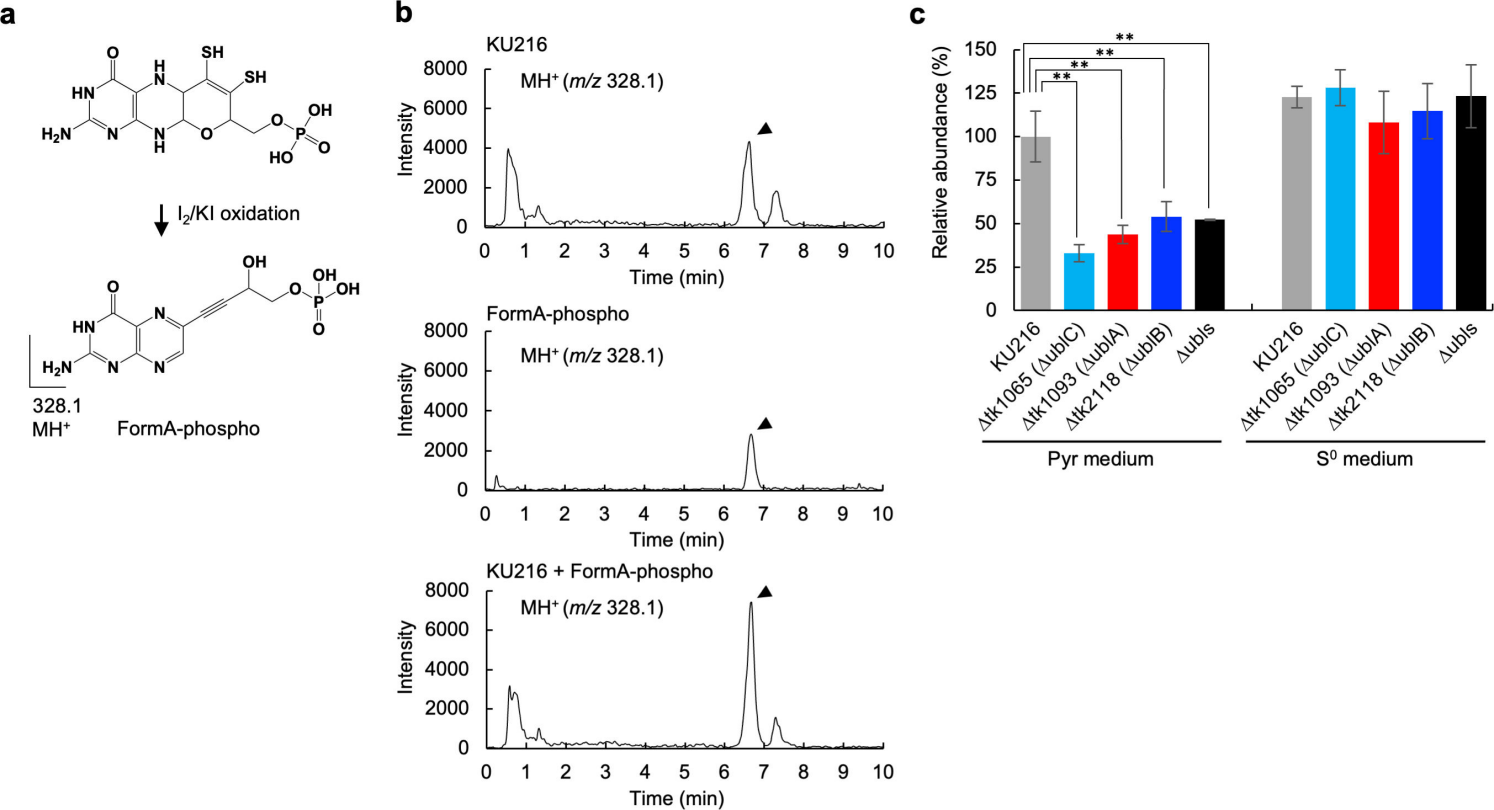
Quantification of the amount of molybdopterin in cells. (a) Molybdopterin was oxidized by iodine, and the derivatized FormA-phospho was detected and quantified by LC-MS. (b) Mass chromatograms of monovalent positive ions of FormA-phospho (*m*/*z* 328.1) extracted from *T. kodakarensis* KU216 cells grown in Pyr medium (upper) and extracted from xanthine oxidase for reference (middle). Co-injection analyses of the above (lower). FormA-phospho was eluted around 6.7 min and is indicated by an arrowhead. (c) Normalized amounts of FormA-phospho in each sample are shown. The mass spectrometry peak area values for FormA-phospho were normalized against their corresponding protein amounts in cell extracts. The value of the KU216 strain in Pyr medium was set to 100%. Means ± standard deviations of three independent measurements are shown. Statistical significance among the relative abundances of KU216 and the Ubl-deficient strains was determined using the Dunnett test. **P < 0.01.

We next measured the quantity of MPT in the cell. MPT in the cell extract from each strain was oxidized to FormA-phospho by iodine under acidic conditions (Fig. 3a) (5, 27, 28) then subjected to liquid chromatography with mass spectrometry (LC-MS) analysis. As shown in Fig. 3b, the proton adduct ion identical to FormA-phospho (*m*/*z* 328.1) was detected at ~6.7 min and further confirmed by co-injection analysis with FormA-phospho prepared from bovine xanthine oxidase (29) (Fig. 3b). Next, the quantity of FormA-phospho in each Ubl-deficient strain and the ∆ubls strain was quantified (Fig. 3c). When cultivated in Pyr medium, approximately half the amount of FormA-phospho was detected in these mutants compared with the parent strain. However, in S^0^ medium, the levels of FormA-phospho in each Ubl-deficient strain were completely restored (Fig. 3c). These results indicate that all Ubls are involved in Wco biosynthesis under sulfur-poor conditions but are dispensable for MPT biosynthesis under sulfur-rich conditions.

**Fig 4:**
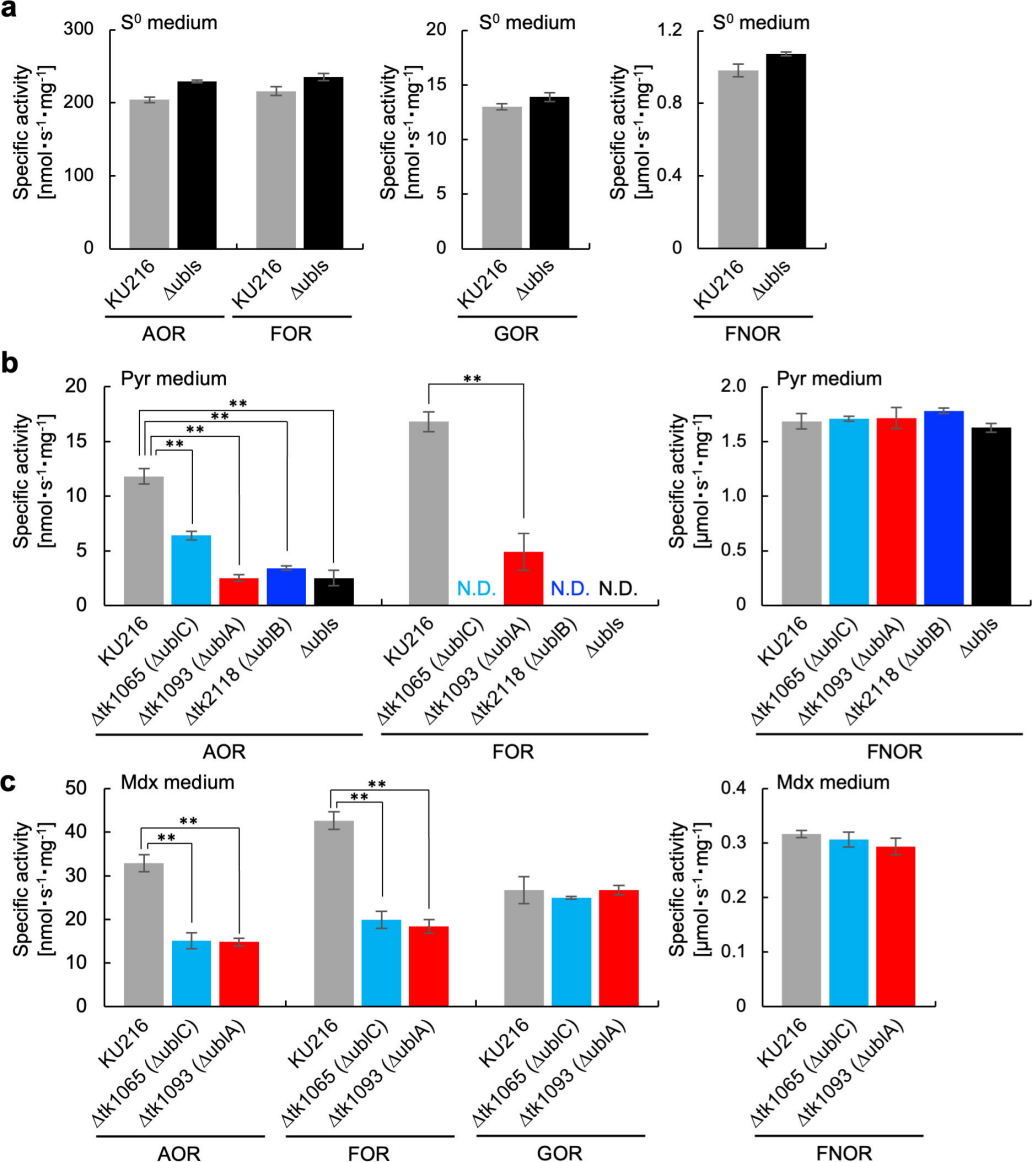
Activities of Wco-containing enzymes and FNOR in Ubl-deficient strains of *T. kodakarensis*. The activities of Wco-containing enzymes and FNOR in cell extracts prepared from cells grown in S^0^ (**a**), Pyr (**b**), and Mdx (**c**) media were measured at 85°C. Values are averages of triplicate experiments, and their statistical significance among the relative abundances of KU216 and the Ubl-deficient strains was determined using the Dunnett test. **P < 0.01. FNOR, ferredoxin:NADP^+^ oxidoreductase; FOR, formate:ferredoxin oxidoreductase; N.D., not detected.

Furthermore, we measured the activities of Wco-containing oxidoreductases AOR, formate:ferredoxin oxidoreductase (FOR), and GOR in the cell lysates of Ubl-deficient strains grown in three different media (S^0^ medium, Pyr medium, and Mdx medium; Fig. 4a through c). No differences were observed in the activities of the three Wco-containing enzymes in parent and ∆ubls strains grown in S^0^ medium (Fig. 4a). These results were consistent with the result that the levels of MPT derivatives in the ∆ubls strain and each Ubl-deficient strain were comparable to that of the parent strain (Fig. 3). In Pyr medium, the AOR activities of the ∆ublA, ∆ublB, and ∆ubls were less than half of those of the parent strain. Also, the AOR activity of ∆ublC was ~54% of that of the parental strain. The FOR activity of ∆ublA was less than half of that of the parent strain, and no FOR activity was detected for ∆ublC, ∆ublB, and ∆ubls. GOR activity could not be determined because the activity was below the detection limit (Fig. 4b). By contrast, GOR activity was enhanced in Mdx medium (25), and the GOR activities of ∆ublC and ∆ublA strains were almost the same as that of the parent strain, suggesting that UblC and UblA are not involved in the maturation of active GOR (Fig. 4c). As a control, the activity of ferredoxin:NADP^+^ oxidoreductase (FNOR), which does not contain Wco and instead contains an iron-sulfur cluster (30) formed without Ubls, was also measured. The FNOR activity of each Ubl-deficient strain was comparable to that of the parent strain under all culture conditions (Fig. 4a through c). These results suggest that Wco in GOR is biosynthesized only by a specific Ubl (UblB) under S^0^-poor conditions.

### UblA is required for efficient s^2^U formation in tRNAs

Ubl, belonging to the ThiS family, is involved in the formation of 2-thiolated uridine derivatives at positions 34 and 54 of tRNAs (31–33). In *T. kodakarensis* tRNAs, m^5^s^2^U is located at position 54 (34), but the chemical structure of the 2-thiolated uridine derivative at position 34 is still unclear. Therefore, we investigated the 2-thiolated uridine derivative in *T. kodakarensis* before evaluating the involvement of Ubls in 2-thiolated uridine formation. The tRNA fraction prepared from the parent strain of *T. kodakarensis* was digested into nucleosides and subjected to liquid chromatography-tandem mass spectrometry (LC-MS/MS) analysis. The proton adduct ion of 5-methyl-2-thiouridine (m^5^s^2^U, *m*/*z* 275.1) was observed at ~10.3 min (Fig. S4a), consistent with a previous report showing m^5^s^2^U located at position 54 in tRNA of *T. kodakarensis* (34). In addition, we successfully detected 5-cyanomethyl-2-thiouridine (cnm^5^s^2^U, *m*/*z* 300.1 at ~9.7 min) and its precursor 5-cyanomethyluridine (cnm^5^U, *m*/*z* 284.1 at ~13.2 min) with confirmation by MS^2^ analysis and co-injection analysis with digested tRNA of *Methanocaldococcus jannaschii*, in which cnm^5^s^2^U is attached to position 34 of tRNA(35) (Fig. S4a through c).

**Fig 5:**
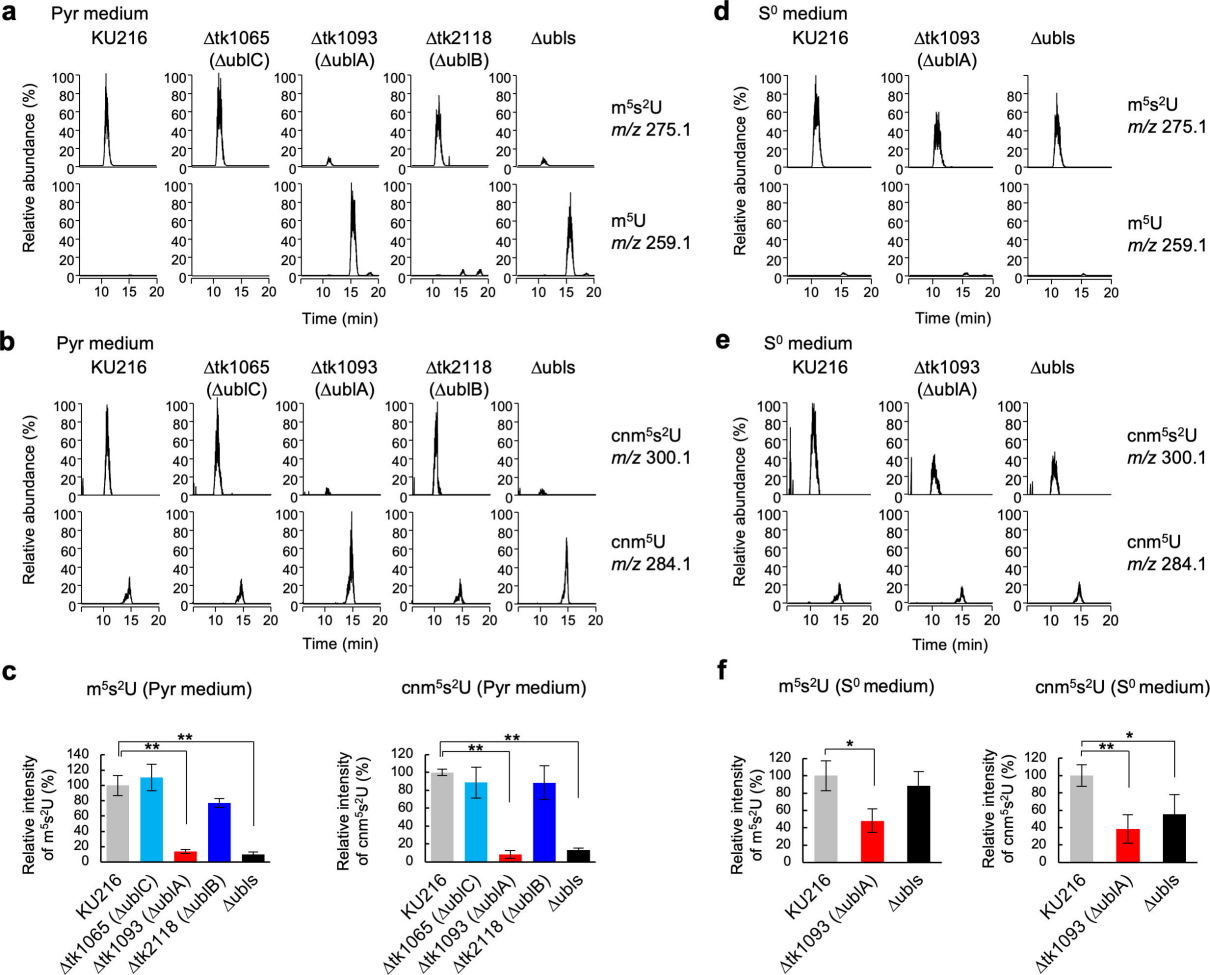
LC-MS/MS analysis of thiolated nucleosides in tRNA. The mass chromatograms show monovalent positive ions of the modified nucleosides of m^5^s^2^U (*m*/*z* 275.1) and m^5^U (*m*/*z* 259.1), cnm^5^s^2^U (*m*/*z* 300.1), and cnm^5^U (*m*/*z* 284.1) in total tRNAs prepared from cells of the *T. kodakarensis* KU216 strain, Ubl-deficient strains, and the ∆ubls strain grown in the absence (**a and b**) or presence (**d and e**) of S^0^. Relative abundance represents the relative intensity of each peak normalized by the intensity of 1-methylinosine. The values of m^5^s^2^U and cnm^5^s^2^U in KU216 strain were set to 100%, respectively. For m^5^U and cnm^5^U, the values in ∆ublA strain in Pyr medium was set to 100%. The results of LC-MS/MS analyses (**a, b, d, and e**) are displayed as bar graphs (**c and f**). Relative abundances of m^5^s^2^U and cnm^5^s^2^U in KU216 (gray), ∆ublC (cyan), ∆ublA (red), ∆ublB (blue), and ∆ubls (black) strains grown in the absence (**c**) or presence (**f**) of S^0^ are shown. Means  ±  standard deviations of three independent measurements are shown. Statistical significance among the relative abundances of KU216 and the Ubl-deficient strains was determined using two-sided Student’s *t*-test. *P < 0.05, **P < 0.01.

Next, we investigated the involvement of the *T. kodakarensis* Ubls in the biosynthesis of the two thionucleosides. Total tRNA fractions prepared from Ubl-deficient strains and the ∆ubls strain cultivated at 85°C in Pyr medium were subjected to LC-MS/MS analysis. In the parent strain, m^5^s^2^U and cnm^5^s^2^U were clearly detected (Fig. 5a through c). However, the levels of these thionucleosides in the ∆ublA strain were drastically reduced to ~14% and ~8%, respectively, with the elevation of their precursors, m^5^U and cnm^5^U. The levels of m^5^s^2^U and cnm^5^s^2^U were not altered in ∆ublC and ∆ublB strains (Fig. 5a through c). In the ∆ubls strain, levels of these were also decreased, as observed in the ∆ublA strain (Fig. 5a through c). Conversely, in S^0^-containing ASW-YT-S^0^ medium, the thiolation levels of m^5^s^2^U and cnm^5^s^2^U in ∆ublA and ∆ubls strains were partially but significantly recovered (Fig. 5d through f). In addition to cnm^5^s^2^U and m^5^s^2^U, four thiolated nucleosides (4-thiouridine, s^4^U; 2-methylthio-*N*^6^-hydroxy-norvalylcarbamoyladenosine, ms^2^hn^6^A; 2-thiocytidine, s^2^C; and 2-methylthio-*N*^6^-threonylcarbamoyladenosine, ms^2^t^6^A) were also identified based on mass spectrum data and retention time (35). The levels of these four thionucleosides were comparable between parent and ∆ubl strains (Fig. S5). These results clearly indicate that UblA is the only sulfur-carrier Ubl responsible for s^2^U formation in *T. kodakarensis* tRNA and is required for efficient 2-thiolation of m^5^U and cnm^5^U under sulfur-poor conditions, although UblA is partially dispensable for modifications under sulfur-rich conditions.

### Three Ubls conjugate to specific proteins in *T. kodakarensis*

We next explored whether the three Ubls are able to form protein conjugates, as observed in other organisms (8, 18, 36), in addition to their sulfur-carrier roles. Similar to eukaryotic ubiquitin, Ubls in bacteria also covalently attach to the Lys residues of target proteins via their C-terminal Gly residues. To this end, whole soluble proteins from cells grown in S^0^-containing medium were separated by sodium dodecyl-sulfate polyacrylamide gel electrophoresis (SDS-PAGE) and subjected to Western blotting with each Ubl-specific antibody (Fig. S6). In the parent strain, many signals indicating Ubl-conjugating proteins were observed, along with signals corresponding to monomeric Ubls at ~10 kDa [theoretical mass: UblC (TK1065), 9.1 kDa; UblA (TK1093), 7.2 kDa; and UblB (TK2118), 10.1 kDa], whereas these bands were absent in the deletion mutants. Moreover, the major bands observed were also detected in lysates prepared from Ubl-deficient strains cultured in the absence of S^0^, suggesting that Ubls are conjugated to specific proteins, irrespective of the presence or absence of S^0^. In addition, the amount of each Ubl-conjugation band tended to be greater in the Pyr medium than in the S^0^-containing medium. Because there are different gene expression patterns in the presence or absence of S^0^ (17), the Ubl-conjugation pattern may also differ.

## DISCUSSION

**Fig 6:**
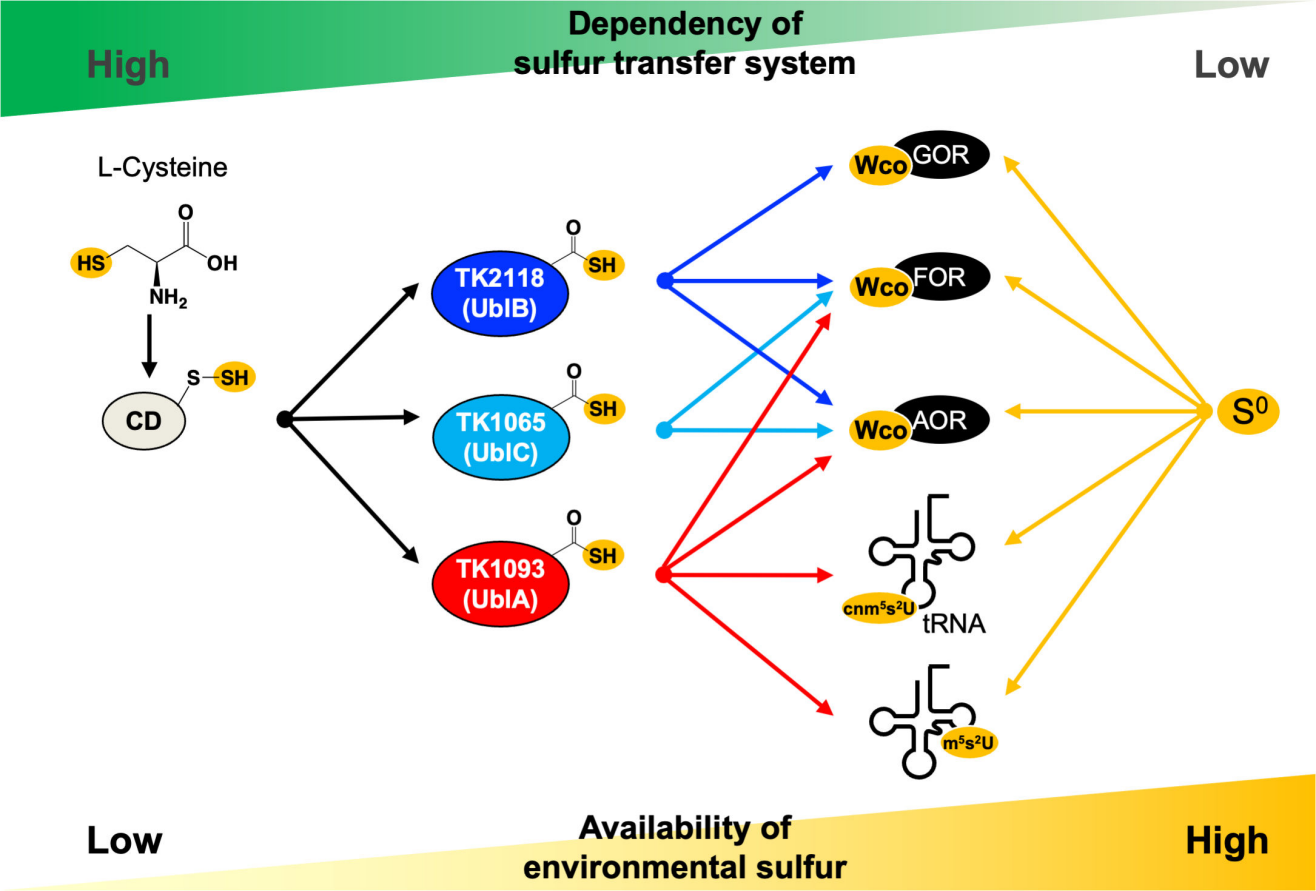
Proposed sulfur-transfer scheme for Wco and tRNA thionucleosides in *T. kodakarensis*. Arrows indicate sulfur flow to the target proteins or tRNA nucleosides. *T. kodakarensis* utilizes two alternative mechanisms, depending on environmental conditions, such as the absence or presence of S^0^.

Here, we investigated the cellular functions of three Ubls in *T. kodakarensis* and revealed the functional versatility and essentiality of these ancient Ubls in the biosynthesis of MPT/Wco and tRNA thiouridine (outlined in Fig. 6), as well as their roles in post-translational modification. Wco in GOR is specifically synthesized via UblB (TK2118), although all three Ubls are redundantly involved in Wco biosynthesis in several oxidoreductases except GOR. For tRNA thiouridine synthesis, only one Ubl (UblA, TK1093) serves as sulfur carrier for both cnm^5^s^2^U and m^5^s^2^U.

In general, different Ubls function strictly in their cognate pathways for sulfur-containing biomolecules (1, 8). Ubls belonging to the ThiS family are relatively shorter in length (~70 amino acid residues) than those in the MoaD family (~80 amino acid residues, Fig. 1d) and have a simpler topology with fewer secondary structural elements (Fig. 1e). Ubls belonging to MoaD and ThiS families in bacteria are essential for sulfur transfer in MPT synthesis (37–39) and tRNA thiolation (12, 18), respectively. In eukaryotes, MOCS2A and Urm1 are required for the biosynthesis of Moco (40, 41) and tRNA thiouridine (42, 43), respectively. Herein, we showed that the ThiS family protein UblA is involved in Moco biosynthesis, in addition to thiouridine synthesis and protein conjugation, implying functional redundancy of an ancient-type Ubl and modest substrate specificity between Ubls and their interaction partners. Related functional redundancy of sulfur-carrier proteins has been reported for the biosynthesis of 2-thiosugar-containing antibiotic secondary metabolites in Actinobacteria; the reaction of 2-thioglucose-6-phosphate synthase BexX of *Amycolatopsis orientali* borrows sulfur-carrier proteins for primary metabolites including MPT (44).

Interestingly, our study clearly revealed flexible utilization of sulfur sources in *T. kodakarensis*; in the presence of environmental S^0^, all Ubls were partially dispensable for the syntheses of these sulfur-containing biomolecules. We previously reported that impairment of iron-sulfur cluster biosynthesis due to the loss of CD was restored in the presence of environmental S^0^ (5). Therefore, we propose that sulfur atoms for sulfur-containing biomolecules in *T. kodakarensis* are supplied from a sulfur-transfer system involving Ubl and CD or from environmental S^0^. Intriguingly, our study also revealed another layer of complexity for the sulfur donor in *T. kodakarensis*; almost 50% of MPT derivative in cells (Fig. 3c) and ~10% of m^5^s^2^U (Fig. 5c) in tRNA were estimated to be biosynthesized through a Ubl-independent sulfur-transfer pathway, even in the absence of S^0^, suggesting the existence of unknown sulfur sources such as S^2−^ equivalents.

We speculated sulfur-transfer pathways for the biosynthesis of MPT/Wco and tRNA thionucleosides in *T. kodakarensis* based on homology searches of the enzymes in these pathways identified in other model organisms (Fig. S7). There is only one E1/MoeB/ThiF family adenylyltransferase homolog (TK2117) in the *T. kodakarensis* genome, and TK2117 presumably activates the glycine residue at the C-terminus of each Ubl in the Wco and thiouridine biosynthesis pathways. There are several reports that one E1/MoeB/ThiF family adenylyltransferase can activate multiple Ubls (4, 18, 40, 41). Our current results showed a decrease in MPT-derivative levels in each Ubl-deficient strain and therefore low or no AOR/FOR activity (Fig. 3, 4b and c). On the other hand, the ∆ublB strain showed growth impairment in Mdx medium (Fig. 2c), where GOR activity is required for growth. Therefore, UblB seems to be a major sulfur-carrier protein for Wco biosynthesis in GOR, implying the existence of an unknown mechanism for specific incorporation of Wco into certain target protein(s) with cognate Ubl(s). Molybdopterin might receive its sulfur atoms after apo-cofactor has bound to the target protein. UblB is the only Ubl that can activate GOR, while all three Ubls can activate FOR and AOR. In plants, Cnx6 (MPT synthase) and Cnx1 (MPT:Mo transferase), which are responsible for two successive steps in Moco biosynthesis, reportedly form a multiprotein complex (45). Such a complex may contribute to secure and specific targeting of the cofactor to the corresponding apo-enzymes, although the mechanism that links specific Ubls and their corresponding apo-enzymes is not known.

For tRNA thiolation, the sulfur atom of UblA thiocarboxylate is likely transferred to tRNAs by sulfurtransferases for m^5^s^2^U54 and cnm^5^s^2^U34 syntheses (Fig. S7). *T. kodakarensis* has two possible 2-thiolase-encoding genes, *ttuA* (*tk1556*) and *ncs6*/*ncsA* (*tk1821*) (46), suggesting that their encoded enzymes recognize and catalyze the modification at positions 34 and 54, respectively, with only one Ubl, UblA (Fig. S7b). We previously showed that TtuB, a homolog of UblA in *Thermus thermophilus*, is a sulfur-carrier Ubl responsible for 2-thiolation at position 54 of tRNA (12). Meanwhile, others reported that SAMP2, a homolog of UblA in *Haloferax volcanii*, is a sulfur-carrier Ubl responsible for 2-thiolation at position 34 of tRNA^Lys^ (33). By contrast, our current study demonstrated for the first time that one sulfur-transfer pathway has two target sites on tRNA, and *in vitro* characterization of the molecular basis of this unique pathway, including putative RNA sulfurtransferases, is under way. We also found that the ∆ublA strain showed growth retardation in Pyr or Mdx media at both optimal and higher temperatures. Since 2-thiolation at positions 34 and 54 of tRNA contribute crucially to decoding accuracy in translation and structural stability of tRNA, respectively (47), significant reductions in the frequencies of these modifications in ∆ublA (Fig. 5a through c) should induce abnormal translation and instability of tRNA, thus resulting in growth retardation and temperature sensitivity. Especially in thermophiles, tRNAs are excessively decorated with various modifications to stabilize the characteristic L-shaped structure (48, 49). Of note, some tRNA modifications, including 2-thiolation of U54, are introduced in a temperature-dependent manner to adapt the structural rigidity and flexibility of tRNA for appropriate protein synthesis at a given temperature (12, 50). Therefore, direct use of sulfide by 2-thiolase would enable faster adaption to a sudden temperature change without a sulfur-transfer system. The direct utilization of sulfide by sulfurtransferases may just be a remnant of protobionts, but for *T. kodakarensis*, which lives in solfatara where acute changes in sulfur concentration and temperature occur, being able to respond to sudden changes in environmental conditions is a great advantage (see below).

Organisms that inhabit inorganic sulfur compound-rich environments are the most likely to have S^0^ reducibility and/or sulfite reductase orthologs and do not have CD orthologs; hence, they probably utilize sulfide (5, 51). For example, the methanogenic archaeon *Methanococcus maripaludis*, which grows in sulfide-rich environments (52), uses sulfide directly for tRNA thiolation and iron-sulfur cluster biosynthesis without CD and Ubls (9, 53). Iron-sulfur cluster synthesis can proceed *in vitro* on an apoprotein in the presence of iron salts and sulfide at millimolar concentrations under anaerobic conditions (54, 55). *T. thermophilus* TtuA can use sulfide as sulfur donor for *in vitro* s^2^U formation (31) without *in vivo* sulfur donor TtuB (12). The C-terminus of *Escherichia coli* MoaD could be thiocarboxylated by sulfide at millimolar concentration, in which case sulfide may be able to support MPT formation *in vitro* (56, 57). A Ubl-mediated sulfur-transfer pathway would compensate the disadvantage of S^0^-dependency, which is susceptible to fluctuation of environmental S^0^, providing an opportunity to survive in a low or no sulfur environment. CD in *T. kodakarensis* likely serves as sulfur donor for Ubls. Our analysis also revealed that all Ubls in *T. kodakarensis* form protein conjugates with specific targets (Fig. S6). It is possible that these post-translational modifications could regulate both MPT and tRNA thiouridine biosynthesis, although further analysis including the identification of target proteins is required. In *H. volcanii*, the Ubls SAMP1 and SAMP2 are essential for Moco biosynthesis and tRNA thiolation, respectively, and these are attached through isopeptide bonds to lysine residues on each protein target, including MoaE and MoeB in Moco biosynthesis (4, 10). Furthermore, in *T. thermophilus*, TtuA and TtuC enzymes for thiouridine synthesis are post-translationally modified by the cognate Ubl TtuB (36).

*T. kodakarensis* probably modulates sulfur-transfer pathways by sensing the fluctuation of S^0^ concentration in the environment, as demonstrated in the present study, and *T. kodakarensis* is also known to switch respiration mode in response to S^0^ availability (58–60). It is believed that protobionts evolved under thermal and sulfur-rich conditions similar to the environments of present hydrothermal vents and volcanic marine sediments (61, 62). Protobionts likely rely on direct utilization of inorganic sulfurs in the biosynthesis of sulfur-containing molecules; therefore, extant hyperthermophiles presumably possess a similar primitive sulfur assimilation mechanism that is dependent on inorganic sulfur. Our characterization of a sulfur-transfer system involving Ubls in *T. kodakarensis* provides insight into an evolutional snapshot of a sulfur assimilation pathway which might have allowed organisms that lived only in solfataric environments to advance into lower-sulfur environments.

## MATERIALS AND METHODS

### Strains and media

Strains of *T. kodakarensis*, KU216 (∆*pyrF*), DAD (∆*pyrF*, ∆*pdaD*), and their derivatives (Table S1) were cultivated at 85°C, 90°C, or 93°C in nutrient-rich ASW-YT-based medium, with addition of 2 g/L S^0^ (ASW-YT-S^0^: S^0^ medium), 2 g/L pyruvate (ASW-YT-Pyr: Pyr medium) (63), or 2 g/L maltodextrin (Amycol number 3 L, Nippon Starch Chemical; ASW-YT-Mdx: Mdx medium). Strain DAD, disruptant of the arginine decarboxylase gene (*pdaD*), displays an agmatine auxotrophy that can be complemented by the *pdaD* gene *in trans*, enabling agmatine-based selection of transformants (64). For selection of a series of disruptants, ASW-AA-S^0^ medium composed of ASW, vitamin mixture, modified Wolfe’s trace minerals, 20 amino acids, and 2 g/L S^0^ was used (63, 65). Gelrite (1 wt/vol %, Wako) was added to ASW-YT-based medium with polysulfide (10 g Na_2_S 9H_2_O and 3 g sulfur flowers in 15 mL H_2_O) for plates. All chemicals and reagents were of analytical grade.

### Construction of gene deletants in *T. kodakarensis*

Specific gene disruptions through double-crossover homologous recombination were performed in *T. kodakarensis* as described previously (63, 66). The disruption vector for *tk1065*, *tk1093*, and *tk2118*, 936,859–936,611 (−) bp, 962,317–962,520 (+) bp, and 1,900,501–1,900,235 (−) bp in the *T. kodakarensis* genome, respectively, were constructed. Briefly, the 5′-flanking and 3′-flanking regions (each ~1,000 bp) of each *ubl* gene were separately amplified with a set of primers (Table S2) by PCR using *T. kodakarensis* genomic DNA. The DNA fragments containing 5′-flanking and 3′-flanking regions for *tk1065* and *tk2118* disruption were integrated into *Eco*RI/*Xba*I sites or *Eco*RI/*Kpn*I sites of the pUD2 vector (63) by Gibson assembly, resulting in plasmids pUD2-∆TK1065 and pUD2-∆TK2118, respectively. The DNA fragments containing 5′-flanking, *pdaD* gene cassette, and 3′-flanking regions for *tk1093* disruption were integrated into *Eco*RI/*Xba*I sites of the pUD2 vector by Gibson assembly, resulting in plasmid pUD2-∆TK1093::pdaD. The host strain used for gene disruption was *T. kodakarensis* DAD (∆*pdaD* and ∆*pyrF*) (64). Each mutant was obtained by gene knock-out (for *tk1065* and *tk2118*) or by pop-in/pop-out gene replacement with the *pdaD* gene (for *tk1093*) from the parent strain step-by-step. Gene deletions and replacements were confirmed by DNA sequencing.

### Complementation of the *tk2118* deletion mutant

Primers Tk2118-Fw3 and Tk2118-Rv3 were used to clone *tk2118*. PCR-amplified DNA fragments of the *tk2118* gene were ligated to the pCSG (67) fragment, which was PCR-amplified using primer set pCSG-Fw/pCSG-Rv, yielding the pCSG-TK2118 plasmid. The QuickChange site-directed mutagenesis method (Qiagen) was used to generate Tk2118 mutants with primer set Tk2118∆GG-Fw/Tk2118∆GG-Rv. The pCSG-TK2118 and derivative constructs were individually introduced into the *T. kodakarensis* ∆ublB strain (Δ*pdaD*, Δ*pyrF*, and Δ*tk2118*), and transformants were selected by growth in the absence of agmatine. Cells were maintained in ASW-YT-based medium supplemented with 2 g/L Mdx.

### TK2117 enzyme assay

ATP:TK2118 adenylyltransferase reaction was performed in a reaction mixture containing 50 mM Tris-HCl buffer (pH 8.0), 10 mM ATP, 5 mM MgCl_2_, and TK2117 (4.0 µM) in the presence or absence of TK2118 (10.0 µM) at 80°C. Enzyme activity was measured by quantifying the phosphate concentration converted from the pyrophosphate product in the reaction mixture. The amount of phosphate was determined using a Biomol Green phosphate assay reagent kit (Enzo Life Sciences) following addition of 1–2 units of inorganic pyrophosphatase (Sigma-Aldrich) to the reaction mix. One unit was defined as the amount of enzyme that produced 1 µmol phosphate/min.

### Extraction and mass spectrometry analysis of FormA-phospho from *T. kodakarensis*

Wco and MPT in *T. kodakarensis* cell was extracted (5, 27, 28) and quantified by LC-MS as follows. *T. kodakarensis* cells were cultivated to logarithmic phase in 100 mL of Pyr medium or S^0^ medium. Cell pellets were washed twice with 30 mL of 1× ASW, resuspended in 300 µL of 10 mM Tris-HCl (pH 8), sonicated, and centrifuged. The protein concentration in the supernatant was determined by the Bradford method (68). The supernatant was adjusted to pH 2.3 by adding 6 M HCl, 200 µL of a solution containing 1 wt/vol % iodine and 2 wt/vol % potassium iodide was added to the cell lysate, and the lysate was heated at 100°C for 30 min to obtain the MPT fluorescent derivative FormA-phospho. Excess iodine was removed by addition of 50 µL of 100 mM ascorbic acid, and the sample was adjusted to pH 8.0 by adding 6 M NaOH. MPT derivative was also extracted from xanthine oxidase in bovine milk (Sigma-Aldrich) for comparison. The LC-MS system consisted of a Nexera X2 high-performance liquid chromatography system and an LCMS-8060NX triple quadrupole mass spectrometer (Shimadzu). Samples were chromatographed using a Kinetex EVO C18 column (100 × 4.6 mm, GL Science) at a flow rate of 0.5 mL/min with a solvent consisting of 50 mM ammonium acetate (pH 6.8) and 7 vol/vol % methanol, and the eluent was sprayed directly into the mass spectrometer. Ions were scanned in positive polarity mode over an *m*/*z* range of 80–350 throughout the separation. The amount of FormA-phospho in each sample was quantified from the MS peak area, and the value was normalized against the total protein amount in each sample.

### Oxidoreductase enzyme assays

*T. kodakarensis* cells were cultivated to logarithmic phase in the presence of S^0^ (0.2 wt/vol %), then collected and disrupted anaerobically by incubating with a lytic solution containing 2 mM dithiothreitol and 2-mM dithionite in 50 mM Tris-HCl (pH 8) supplemented with 5 U/mL DNase I (Takara Bio). Substrate-dependent reduction of methyl viologen in the cell-free extract was assayed as a change in *A*_603 nm_ to determine tungstopterin enzyme activities, crotonaldehyde for AOR (22), formaldehyde for FOR (23), and glyceroaldehyde-3-phosphate for GOR (24) at 80°C under anaerobic conditions (O_2_ <1 ppm). NADH-dependent reduction of methyl viologen (Sigma-Aldrich) was measured to detect FNOR activity (30). Protein concentrations were determined by the Bradford method with bovine serum albumin as a standard (68). One unit was defined as the amount of enzyme that produced 1 µmol of product/min.

### Preparation and mass spectrometry analysis of total tRNAs from *T. kodakarensis*

*T. kodakarensis* cells were cultivated to logarithmic phase in the presence or absence of S^0^. Total RNA was extracted from harvested cells using the acid guanidinium thiocyanate-phenol-chloroform method (34). After isopropanol precipitation, the RNA solution was mixed with 2-butoxyethanol and purified by ethanol precipitation. Total RNAs were separated by 10 wt/vol % denaturing PAGE containing 7 M urea, and bands corresponding to total tRNAs were excised from the gel stained with 0.05 wt/vol % toluidine blue (Wako). Total tRNAs were eluted from the gel using elution buffer consisting of 0.3 M sodium acetate (pH 5.3), 1 mM EDTA-NaOH (pH 8.0), and 0.1 wt/vol % SDS, and filtered (Ultrafree-MC, HV, 0.45 µm; Merck Millipore). Total tRNAs were recovered by ethanol precipitation and desalted by drop dialysis on a nitrocellulose membrane (MFMillipore, Merck Millipore) against ultrapure water for 2 h. The principles underlying mass spectrometry analysis of nucleosides have been described previously (34, 48). tRNA mixtures were digested with nuclease P1 (Wako) and bacterial alkaline phosphatase (BAP.C75, Takara Bio). The digest dissolved in acetonitrile was applied to a nanoflow high-performance LC-MS/MS system. The solvent system consisted of 5 mM ammonium acetate, pH 5.3 (solvent A) and acetonitrile (solvent B). Nucleotides were chromatographed by a ZIC-cHILIC column (2.1 × 150 mm, Merck Millipore) with a multistep linear gradient of 90%–50% B from 0 to 30 min, 50% B for 10 min, and 50%–90% B from 40 to 45 min, then initialized to 90% B at a flow rate of 100 µL/min. The chromatographic eluent was sprayed directly into the ESI source of a Q Exactive Hybrid Quadrupole-Orbitrap Mass Spectrometer (Thermo Fisher Scientific). Ions were scanned in positive polarity mode over an *m*/*z* range of 110–900 throughout the separation.

### Expression and purification of Ubls and TK2117 proteins

Plasmids used to overexpress the *tk1065*, *tk1093*, *tk2118*, and *tk2117* genes in *E. coli* were constructed. Briefly, PCR-amplified DNA fragments encoding *tk1065*, *tk1093*, *tk2118*, and *tk2117* genes generated using primer set Tk1065-pET-Fw/Tk1065-pET-Rv, Tk1093-pET-Fw/Tk1093-pET-Rv, Tk2118-pET-Fw/Tk2118-pET-Rv, and Tk2117-pET-Fw/Tk2117-pET-Rv were separately cloned into the *Nde*I/*Bam*HI sites of the pET28a plasmid (Merck Millipore). *E. coli* BL21-CodonPlus (DE3)-RIL cells were used for protein expression. The recombinant *E. coli* cells were grown in 100 mL of Luria-Bertani medium containing 100 µg/mL of ampicillin at 37°C until the OD_600_ reached 0.6. After induction with 1-mM isopropyl-β-D-thiogalactopyranoside for 4 h, cells were harvested by centrifugation, resuspended in buffer A containing Tris-HCl (pH 8) and 150 mM NaCl, and disrupted by sonication. The crude extract was incubated at 85°C for 20 min and centrifuged, and recombinant protein was purified from the supernatant to homogeneity (Fig. S8). The supernatant was applied to 5 mL Ni Sepharose high-performance resin (Cytiva) charged with nickel. The column was washed with buffer A containing 10 mM imidazole, and recombinant proteins were eluted with buffer A containing 200 mM imidazole. Imidazole was removed from the protein solution by a PD-10 desalting column (Cytiva). Protein concentrations were determined by the Bradford method with bovine serum albumin as a standard (68).

### Immunoblotting

Extracts (30 µg) prepared from cells grown in S^0^ medium were separated by SDS-PAGE and transferred to polyvinylidene difluoride membranes (ATTO). Immunodetection was performed with antibodies raised against purified TK1065, TK1093, and TK2118 in rabbit and goat anti-rabbit IgG-AP Conjugate (BioRad), and the signal was visualized using an ImageQuant LAS4000 instrument (Cytiva).

## SUPPLEMENTAL MATERIAL

Supplemental figures and tables mbio.00534-24-s0001.pdf Fig. S1 to S8; Tables S1 and S2.

ASM does not own the copyrights to Supplemental Material that may be linked to, or accessed through, an article. The authors have granted ASM a non-exclusive, world-wide license to publish the Supplemental Material files. Please contact the corresponding author directly for reuse.
