# Supplementary material for: Functional redundancy of ubiquitin-like sulfur-carrier proteins facilitates flexible, efficient sulfur utilization in the primordial archaeon Thermococcus kodakarensis
Source: mBio. 2024 Jul 8;15(8):e00534-24. doi: 10.1128/mbio.00534-24 (PMC11323500; doi:10.1128/mbio.00534-24)
Supplement: Supplemental figures and tables — Fig. S1 to S8; Tables S1 and S2. [file mbio.00534-24-s0001.pdf]

Supporting Information

Functional redundancy of ubiquitin-like sulfur-carrier proteins facilitates flexible, efficient sulfur utilization in the primordial archaeon *Thermococcus kodakarensis*

Ryota Hidese, Takayuki Ohira, Satsuki Sakakibara, Tsutomu Suzuki, Naoki Shigi, and Shinsuke Fujiwara

Figure S1. **Distribution of CD and Ubl orthologs in archaea. a**, Distribution of orthologs for CD and Ubl. Protein sequences were obtained from the UniProt database. Each Ubl ortholog is highlighted in blue (MoaD family) or red (ThiS family) based on phylogenic analysis. **b**, Phylogenetic affiliation of archaeal Ubl orthologs and *E. coli* MoaD/ThiS and *T. thermophilus* HB27 Ubl orthologs, inferred using multiple alignment analysis with the MUSCLE algorithm and a maximum likelihood method using MEGAX 10.2.6. The scale bar represents 50% amino acid residue substitution. Bootstrap support values (100 replicates) >40% are shown at nodes. **c**, Ribbon diagrams of the Alphafold models of TkUbls (Fig. 1e) colored with pLDDT values (blue to orange, high confidence to low confidence).

Figure S2. Pyrophosphate production by TK2117 in the presence of TK2118. ATP:TK2118 adenylyltransferase activity of TK2117 (4.0 μM) at 80°C was measured by detecting the absorbance at 620 nm using a Biomol Green phosphate assay kit coupled with inorganic pyrophosphatase. Means ± standard deviations of three independent measurements are shown. Statistical significance between the presence (●) or absence (○) of TK2118 (10.0 μM) was determined using Student's *t*-test (*p <0.01). Specific activity

of ATP:TK2118 adenylyltransferase of TK2117 was 7.02 ± 0.34 μmol/min/mg.

Figure S3. Confirmation of the genomic construct of each *ubl* mutant of *T. kodakarensis*. **a**, PCR fragments obtained from the 5'- and 3'-flanking regions of each Ubl using the corresponding primers were analyzed by agarose gel electrophoresis: Tk1065-Fw1 and Tk1065-Rv2 fo*r tk1065* disruption (Δ, ΔublC strain: 1.66 kbp) and host strain (H, host strain: 1.90 kbp); Tk1093-Fw1 and Tk1093-Rv2 for *tk1093* disruption (Δ, ΔublA: 2.31 kbp) and host strain (H, host strain: 1.86 kbp); Tk2118-Fw1 and Tk2118-Rv2 for *tk2118* disruption (Δ, ΔublB strain: 1.66 kbp) and host strain (H, host strain: 1.92 kbp). **b**, PCR fragments were obtained from the Δubls strain genome using the corresponding primer sets. DNA size markers are shown in lane M.

Figure S4. LC-MS co-injection analysis to identify tRNA thionucleosides in *T. kodakarensis*. **a**, LC-MS analyses of total nucleosides in tRNAs of *T. kodakarensis* (left), *M. jannaschii* (middle), and a mixture of *T. kodakarensis* and *M. jannaschii*. The top panel shows UV_{254} absorbance. Mass chromatograms show proton adducts of cnm^5s^2U (*m/z* 300.1, 9.7 min), cnm^5U (*m/z* 284.1, 13.2 min), and m^5s^2U (*m/z* 275.1,10.3 min), respectively. Vertical axes show relative abundance (%) of each modified nucleoside. **: unidentified peaks. No m^5s^2U modification was reported in *M. jannaschii*. **b**, Mass chromatogram showing the protonated molecular ion (MH^+) and the base-related ion (BH_2^+) of cnm^5s^2U and cnm^5U. **c**, Collision-induced dissociation spectra of the MH^+ (left panels) and BH_2^+ (right panels) of cnm^5s^2U (upper panels) and cnm^5U (lower panels). BH_2^+, -HCN product ions, and other fragment ions with *m/z* 210.03, 194.06, 125.03, and 100.04 are indicated, according to a previous report(1).

Figure S5. Relative intensities of tRNA thionucleosides in *T. kodakarensis*. Bar graphs show the relative intensities (%) of four tRNA thionucleosides (ms^2hn^6A, ms^2t^6A, s^4U, and s^2C) in the *T. kodakarensis* KU216 strain (gray) and the Δubls strain (black) grown in Pyr medium. The intensities are normalized against 1-methylinosine. The value of KU216 is set to 100%. Means ± standard deviations of three independent measurements are shown. Statistical significance among the relative abundances of KU216 and the Ubl-deficient strains was determined using the two-sided Student's *t*-test (*p <0.05).

Figure S6. TkUbls form protein conjugates in *T. kodakarensis*. Crude extracts (30 µg) of cells grown in ASW-YT-S^0 (S^0 medium) or ASW-YT-based medium supplemented with pyruvate (Pyr medium) for mid-log phase at 85°C were separated by SDS-PAGE and analyzed by western blotting using antisera against His-tagged TK1065, TK1093, and TK2118 (upper panels) and Coomassie Brilliant Blue staining (lower panels) as a loading control (H, host strain; Δ, the disruptant corresponding to specific antisera used). Black arrowheads indicate bands derived from corresponding Ubls; white arrowheads indicate marked specific bands of protein conjugates derived from the corresponding Ubls.

Figure S7. Proposed biosynthesis pathways of molybdopterin (a) and thiouridine derivatives (b) in tRNAs in *T. kodakarensis*. a, *T. kodakarensis* cells possess a set of homologous genes for MPT biosynthesis from a precursor GTP(2). The predicted orthologs in *T. kodakarensis* are: TK2225, GTP 3',8-cyclase; TK0354, cyclic pyranopterin monophosphate synthase; TK1065 (UblC), TK1093 (UblA), and TK2118 (UblB), sulfur-carrier subunit (Ubl); TK2115, MPT synthase; TK0544, MPT adenylyltransferase; and

TK1282 and TK0541, MPT molybdenumtransferase. A sulfurtransferase transfers a sulfur atom to the adenylated Ubl, resulting in the formation of a C-terminal thiocarboxylated Ubls. MPT synthase MoaE (TK2115) catalyzes the incorporation of two sulfur atoms from Ubl thiocarboxylate into precursor Z to form MPT, and MPT is further adenylated by MPT adenylyltransferase MogA (TK0544). Tungsten is incorporated into the resultant intermediate by two paralogs of MPT molybdenumtransferase MoeA (TK1282 and TK0541), resulting in the formation of Wco. **b**, The predicted orthologs of thionucleoside biosynthesis in *T. kodakarensis* are: TK2135 is the experimentally validated ortholog of RumA-type m^5U_{54} methyltransferase(3); TK1574 is an ortholog of a radical *S*-adenosylmethionine-dependent methyltransferase from *Methanocaldococcus infernus* Elp3 (MinElp3)(4); TK1556 is an ortholog of a sulfurtransferase from *T. thermophilis* TtuA(5) (for m^5s^2U54); TK1821 is an ortholog of a sulfurtransferase from *H. volcanii* NcsA (for s^2U34)(6).

Figure S8. Purity of recombinant proteins. Five micrograms of each recombinant protein was separated by SDS-PAGE and stained with Coomassie Brilliant Blue. M indicates size markers.

a

Organism	CD ortholog	Ubl ortholog
Thermococcus kodakarensis	TK1990	**TK1065**, **TK1093**, **TK2118**
Haloferax volcanii	HVO_RS05220	HvSAMP1, HvSAMP2, HvSAMP3
Pyrococcus horikoshii	-	PH_RS02725, PH_RS05175, PH_RS04800, PH_RS07545
Thermoplasma volcanium	-	TVG_RS00615, TVG_RS02985, TVG_RS04275
Archaeoglobus fulgidus	AF_RS00930, AF_RS02865	AF_RS00100, AF_RS02805, AF_RS08170, AF_RS10595
Methanocaldococcus jannaschii	-	-
Methanococcus maripaludis	-	MMP_RS03440, MMP_RS07000
Methanothermobacter marburgensis	MTBMA_RS08670	MTBMA_RS01575
Pyrobaculum arsenaticum	-	PARS_RS04175, PARS_RS05080, PARS_RS10525, PARS_RS11990
Sulfolobus acidocaldarius	-	SACI_RS03190, SACI_RS04545, SACI_RS07880
Hyperthermus butylicus	-	HBUT_RS00680, HBUT_RS03065, HBUT_RS05700
Thermus thermophilus	TTC1373, TTC0087	TTC0105 (TtuB), TTC0316, TTC1947, TTC1835
Escherichia coli	SufS, IscS, CsdA	EcThiS, EcMoaD

b

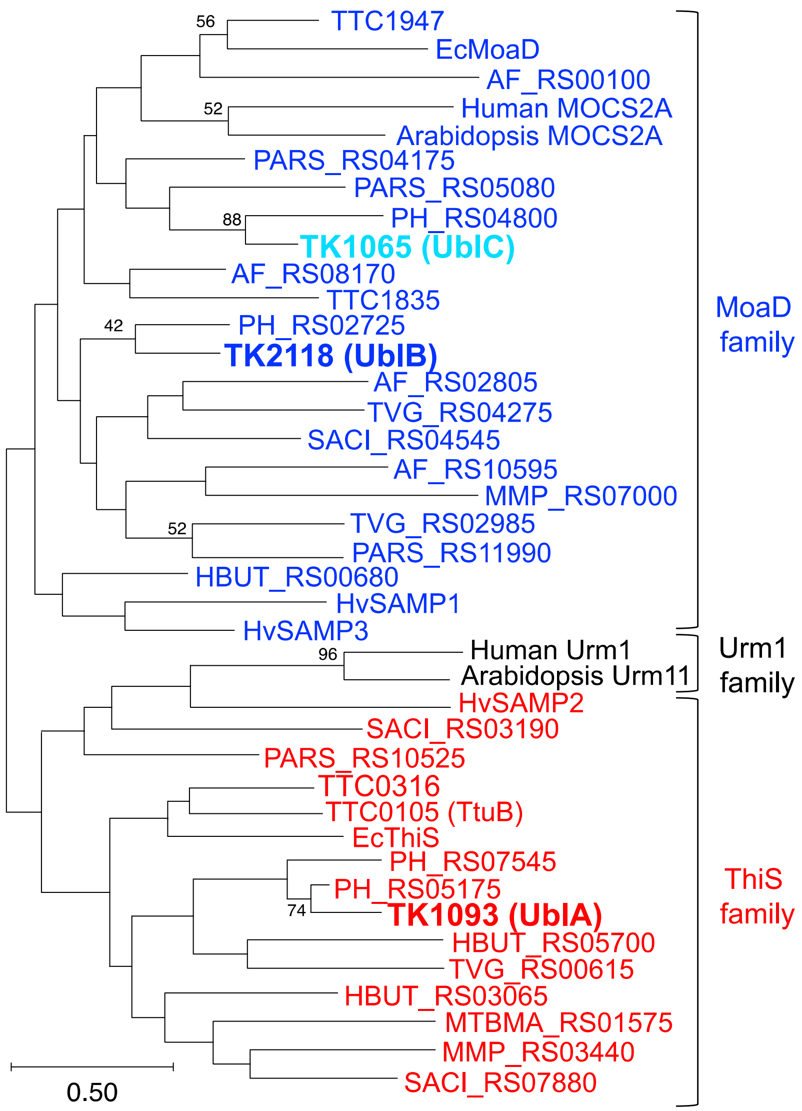

c

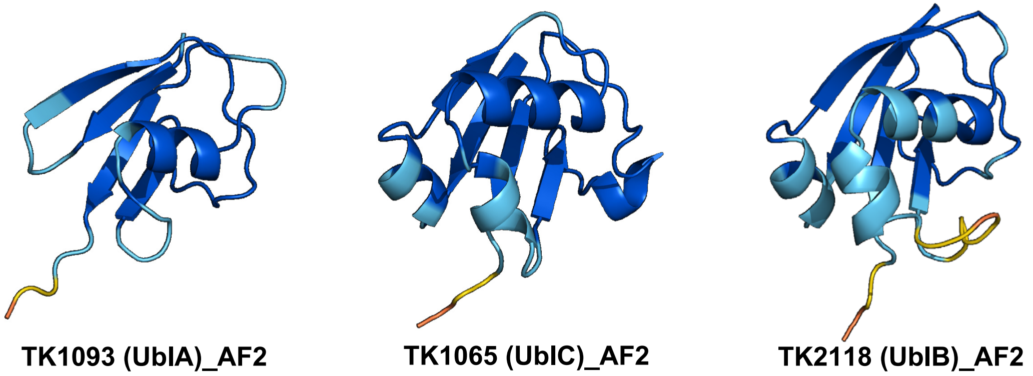

Supplementary Figure S1 Hidese et. al.

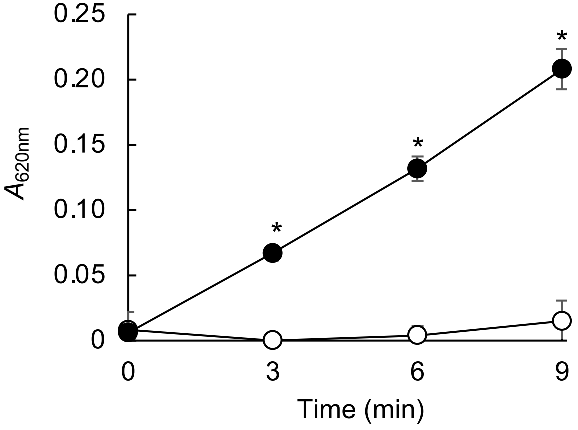

Supplementary Figure S2 Hidese et. al.

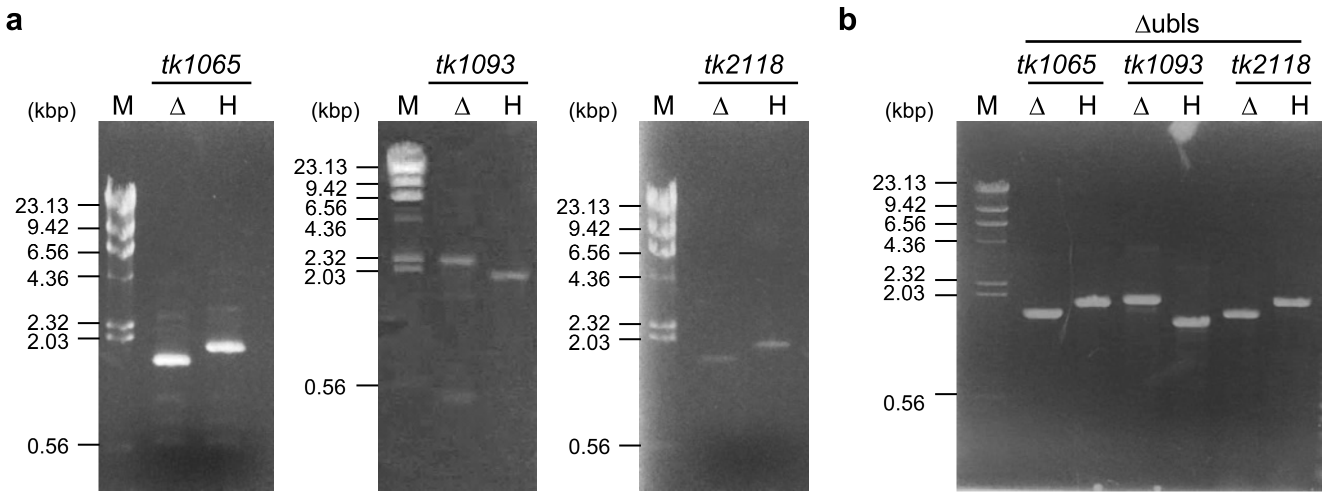

Supplementary Figure S3 Hidese et. al.

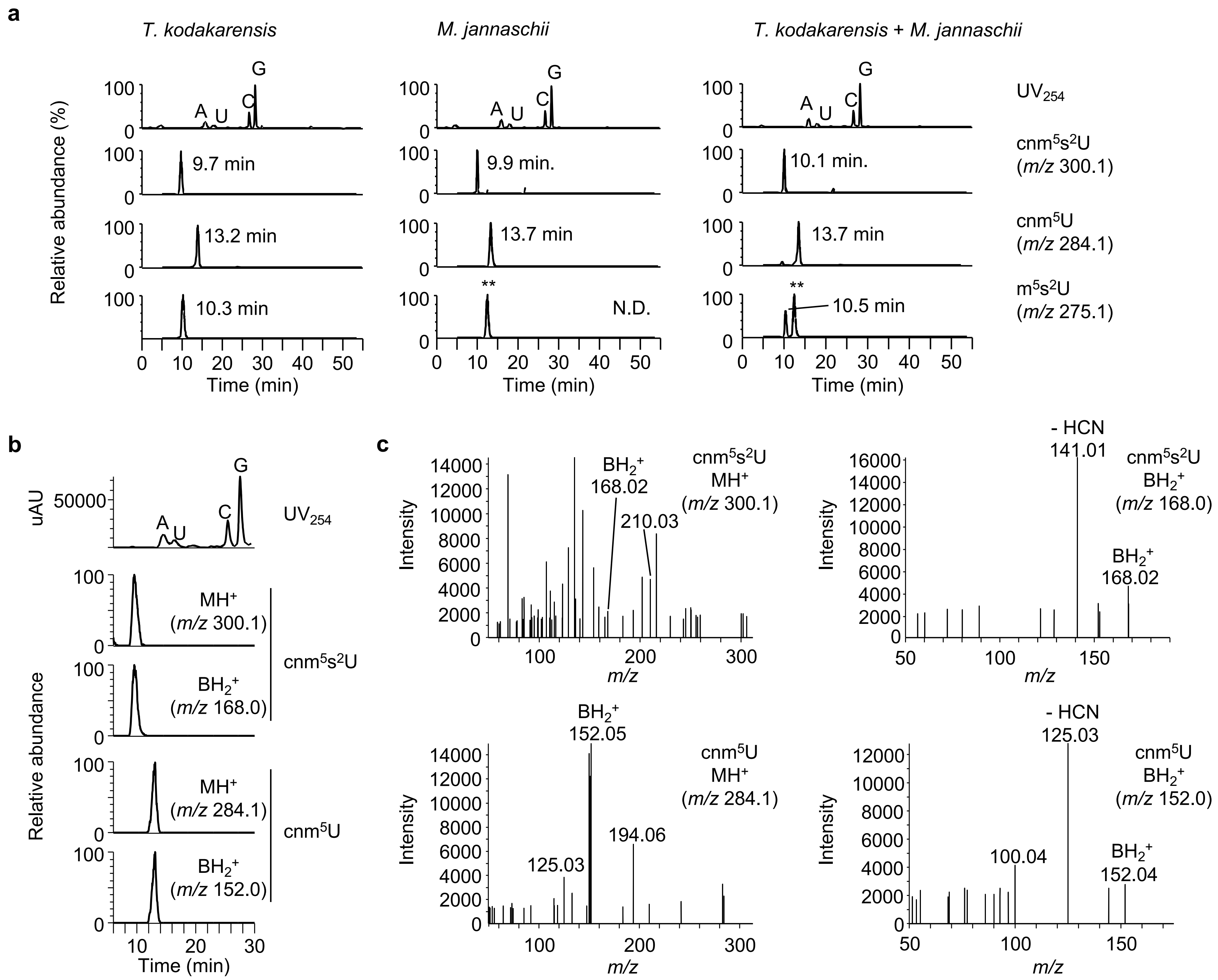

Supplementary Figure S4 Hidese et. al.

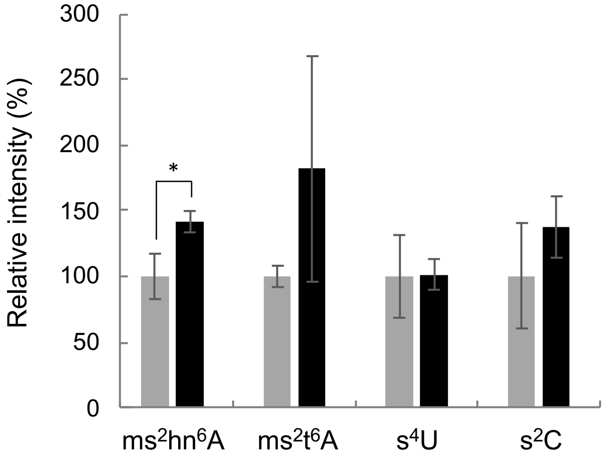

Supplementary Figure S5 Hidese et. al.

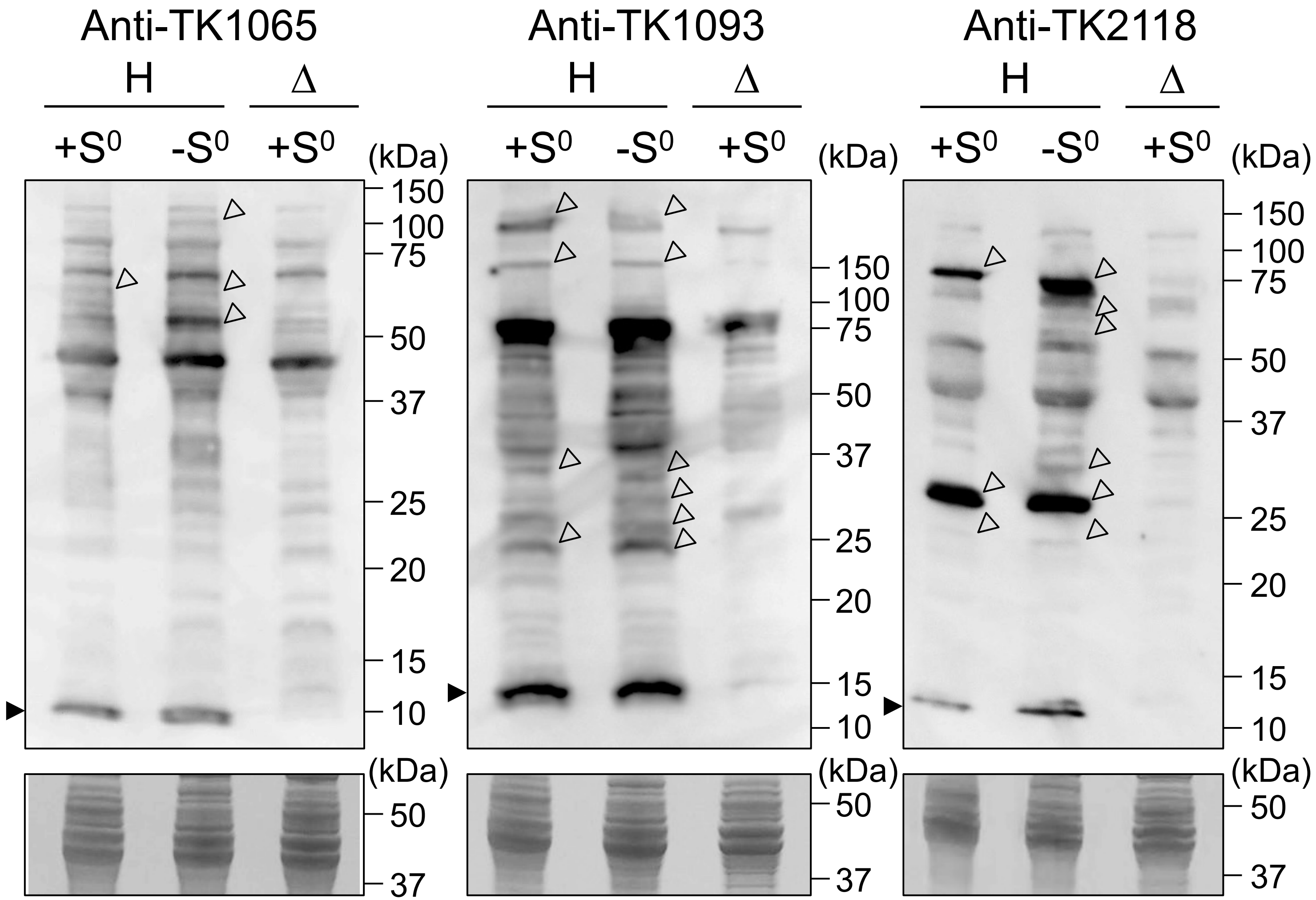

Supplementary Figure S6 Hidese et. al.

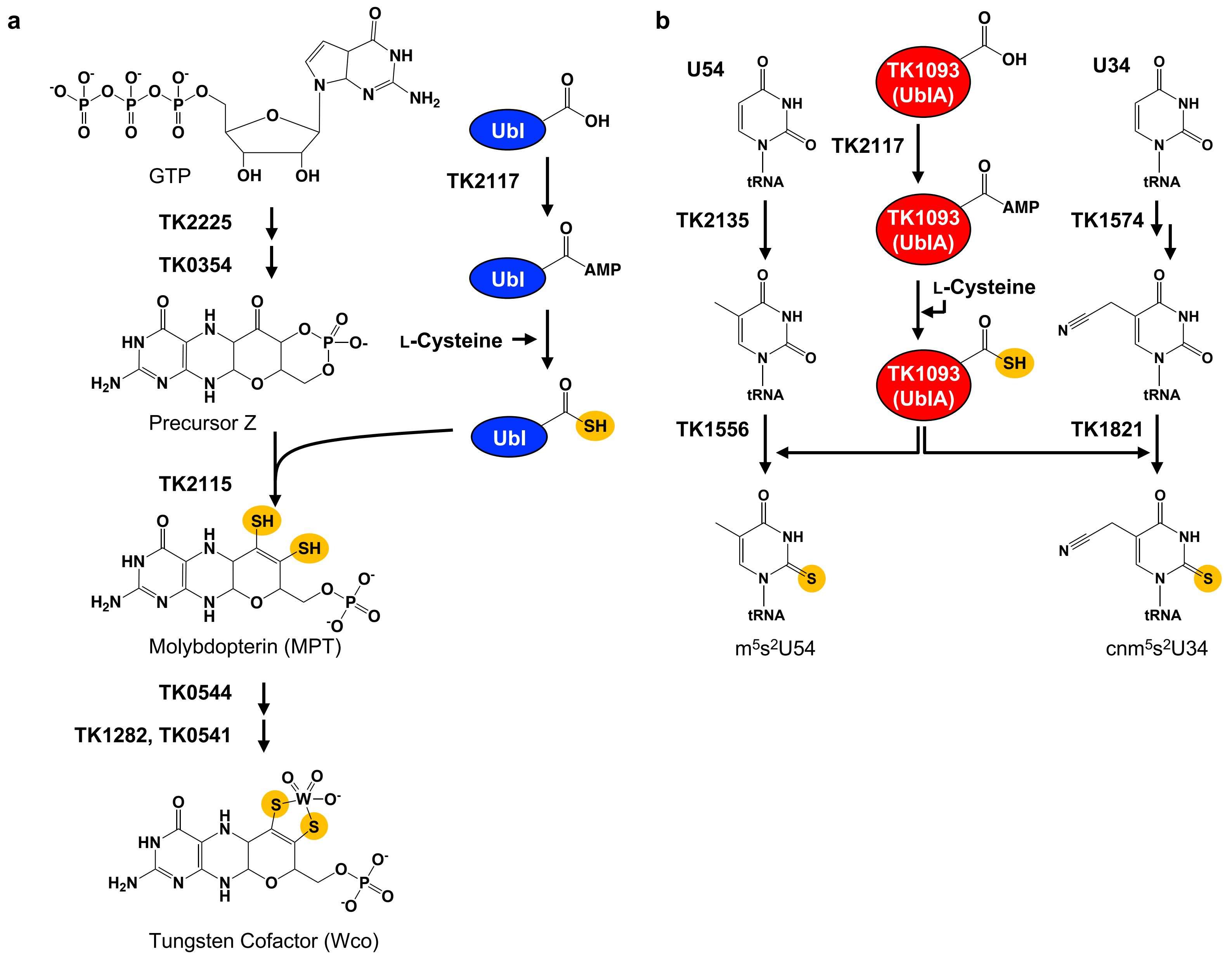

Supplementary Figure S7 Hidese et. al.

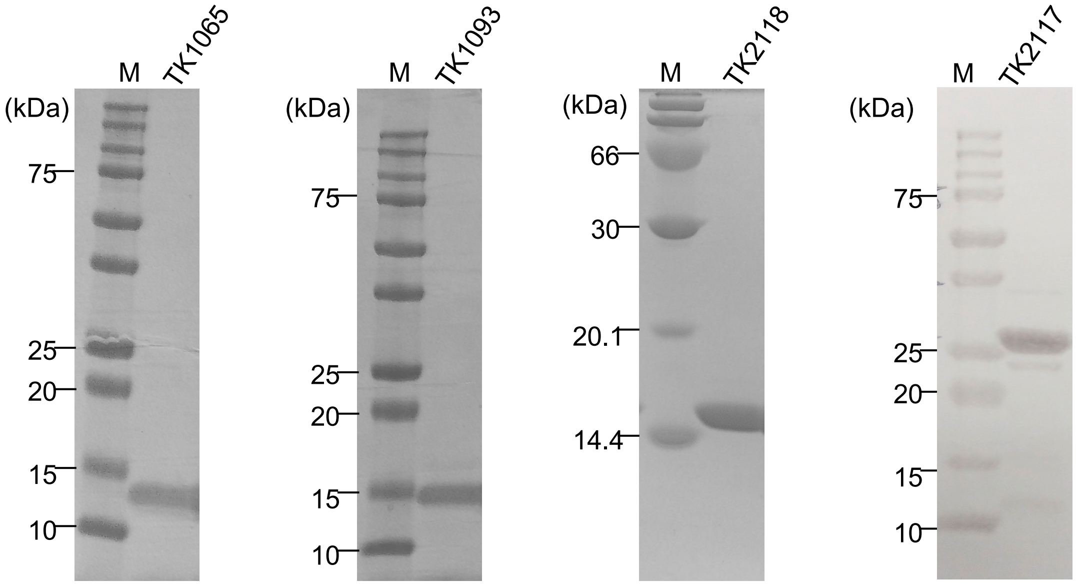

Supplementary Figure S8 Hidese et. al.

Table S1. Strains used in this study

Strains	Relevant characteristics	Source
Escherichia coli		
DH5α	F^{-}, Φ80d*lacZ*ΔM15, Δ(*lacZYA-argF*)U169, *deoR, recA*1, *endA*1, *hsdR*17(r_K^{-}, m_K^{+}), *phoA, supE*44, *λ*$^{-}$, *thi*-1, *gyrA*96, *relA*1	Takara Bio
BL21-CodonPlus(DE3)-RIL	*E. coli* B F^{-} *ompT hsdS*(r_B^{-} m_B^{-}) *dcm*+ Tet^{r} *gal* λ(DE3) *endA* Hte [*argU ileY leuW* Cam^{r}]	Agilent Technologies
Thermococcus kodakarensis		
KU216	*Thermococcus kodakarensis* KOD1 Δ*pyrF*	(7)
DAD	KU216 Δ*pdaD*	(8)
Δtk1065 (ΔublC)	DAD Δ*tk1065*	This study
Δtk1093 (ΔublA)	DAD Δ*tk1093*::*pdaD*	This study
Δtk2118 (ΔublB)	DAD Δ*tk2118*	This study
Δubls	DAD Δ*tk1065,* Δ*tk1093::pdaD,* Δ*tk2118*	This study

Table S2. Primers used in this study

Primer	Sequence (5′ to 3′)
For *tk1065* gene disruption vector construction	
Tk1065-Fw1[a]	CTATGACCATGATTAC*GAATTC*GGTTAACAAGCTCCCGAC
Tk1065-Rv1[a]	AACTTTATTGCCACCGTGAACCACCTTAAA
Tk1065-Fw2[b]	TTTAAGGTGGTTCACGGTGGCAATAAAGTT
TK1065-Rv2[b]	GTTTGACAGCTGGAC*TCTAGA*GGAGGGGTACATGTA
For *tk1093* gene disruption vector construction	
Tk1093-Fw1[a]	CTATGACCATGATTAC*GAATTC*AATGATTTATAACCCTGC
Tk1093-Rv1[a]	GCTAAAACATCATTCTCGCCCTCACCACCTGTTC
Tk1093-Fw2[b]	ATGTTCCCCTACTGAAAAAGGGAAGGGAGAAATC
Tk1093-Rv2[b]	TGACAGCTGGAC*TCTAGA*CCGAGATGAGGGAGG
Tk1093-pdaD-Fw[c]	GAACAGGTGGTGAGGGCGAGAATGATGTTTTAGC
Tk1093-pdaD-Rv[c]	GATTTCTCCCTTCCCTTTTTCAGTAGGGGAACAT
For *tk2118* gene disruption vector construction	
Tk2118-Fw1[a]	TGACCATGATTAC*GAATTC*CACCGTGCTGGTTGCTC
Tk2118-Rv1[a]	CTCTCGGTCAGCATGCTTTTCATCACCGGG
Tk2118-Fw2[b]	CCCGGTGATGAAAAGCATGCTGACCGAGAG
Tk2118-Rv2[b]	GAGGATCCCCG*GGTACC*TCGCCAAGTACACAGAG
For genetic complementation vector construction	
Tk2118-Fw3	AGGAGGTGTTGTGGAGTGAGAATAAAAGTCAGATA
Tk2118-Rv3	GCTCGAGCGGCCGCCATATGTTAACCACCACTTACAG
pCSG-Fw	CATATGGCGGCCGCTCGAGCA
pCSG-Rv	TCCACAACACCTCCTTGG
Tk2118ΔGG-Fw	ATTCCCGCCTGTAAGTTAACATATGGCGGCCG
Tk2118ΔGG-Rv	CGGCCGCCATATGTTAACTTACAGGCGGGAAT
For expression vector construction	
Tk1065-pET-Fw	GTGCCGCGCGGCAGC*CATATG*CTGGTTAAGCTGTTCGC
Tk1065-pET-Rv	GGAGCTCGAATTC*GGATCC*TCAACCGCCTCCCGCCG
Tk1093-pET-Fw	GCCGCGCGGCAGC*CATATG*ATAAGGGTCAAAG
Tk1093-pET-Rv	AGCTCGAATTC*GGATCC*TCATCCGCCAGAGACG
Tk2118-pET-Fw	TGCCGCGCGGCAGC*CATATG*GTGAGAATAAAAGTCAGA
Tk2118-pET-Rv	CGGAGCTCGAATTC*GGATCC*TTAACCACCACTTACAGG
Tk2117-pET-Fw	GTGCCGCGCGGCAGC*CATATG*CTGACCGAGAGAGAAC

Tk2117-pET-Rv	GAGCTCGAATTC*GGATCC*CTACTTAAGTTCCACTAC

Restriction sites are underlined and italicized.

[a]Primer sequence for PCR amplification of the 5′-flanking region of each *ubl*.

[b]Primer sequence for PCR amplification of the 3′-flanking region of each *ubl*.

[c]Primer sequence for PCR amplification of the *pdaD* gene with promoter.

References

1. Yu N, Jora M, Solivio B, Thakur P, Acevedo-Rocha CG, Randau L, de Crécy-Lagard V, Addepalli B, Limbach PA. 2019. tRNA Modification Profiles and Codon-Decoding Strategies in Methanocaldococcus jannaschii. J Bacteriol 201.
2. Yokoyama K, Leimkühler S. 2015. The role of FeS clusters for molybdenum cofactor biosynthesis and molybdoenzymes in bacteria. Biochim Biophys Acta 1853:1335-49.
3. Hirata A, Suzuki T, Nagano T, Fujii D, Okamoto M, Sora M, Lowe TM, Kanai T, Atomi H, Suzuki T, Hori H. 2019. Distinct Modified Nucleosides in tRNA(Trp) from the Hyperthermophilic Archaeon Thermococcus kodakarensis and Requirement of tRNA m(2)G10/m(2)(2)G10 Methyltransferase (Archaeal Trm11) for Survival at High Temperatures. J Bacteriol 201.
4. Selvadurai K, Wang P, Seimetz J, Huang RH. 2014. Archaeal Elp3 catalyzes tRNA wobble uridine modification at C5 via a radical mechanism. Nat Chem Biol 10:810-2.
5. Shigi N, Sakaguchi Y, Suzuki T, Watanabe K. 2006. Identification of two tRNA thiolation genes required for cell growth at extremely high temperatures. J Biol Chem 281:14296-306.
6. Chavarria NE, Hwang S, Cao S, Fu X, Holman M, Elbanna D, Rodriguez S, Arrington D, Englert M, Uthandi S, Söll D, Maupin-Furlow JA. 2014. Archaeal Tuc1/Ncs6 homolog required for wobble uridine tRNA thiolation is associated with ubiquitin-proteasome, translation, and RNA processing system homologs. PLoS One 9:e99104.
7. Sato T, Fukui T, Atomi H, Imanaka T. 2005. Improved and versatile transformation system allowing multiple genetic manipulations of the hyperthermophilic archaeon Thermococcus kodakaraensis. Appl Environ Microbiol 71:3889-99.
8. Fukuda W, Morimoto N, Imanaka T, Fujiwara S. 2008. Agmatine is essential for the cell growth of Thermococcus kodakaraensis. FEMS Microbiol Lett 287:113-20.
